# Supplementary material for: Reconciling Mining with the Conservation of Cave Biodiversity: A Quantitative Baseline to Help Establish Conservation Priorities
Source: PLoS One. 2016 Dec 20;11(12):e0168348. doi: 10.1371/journal.pone.0168348 (PMC5173368; doi:10.1371/journal.pone.0168348)
Supplement: S1 Dataset — (ZIP) [file pone.0168348.s002.zip › Taxa/Serra Norte/SN_2013/N4E/Lista N4E-14.pdf]

CAVIDADE N4E-0014

Classe	Ordem	Fam/Outros	Gên/Outros	Espécie	S-U	U-S	Zon.
Arachnida	Acari	(Parasitengonina)		sp.2		X	E
Arachnida	Acari	Ascidae		sp.3		X	A
Arachnida	Acari	Ixodidae	Amblyomma	*cajennense*	X		
Arachnida	Acari	Ixodidae	Amblyomma	sp.1	X		
Arachnida	Acari	Macronyssidae		sp.1		X	A
Arachnida	Acari	Mesostigmata		sp.1		X	E
Arachnida	Acari	Trombiculidae		sp.39		X	A
Arachnida	Amblypygi	Charinidae	*Charinus*	sp.1		1	A
Arachnida	Amblypygi	Phrynidae	*Heterophrynus*	*longicornis*	X		
Arachnida	Araneae	Corinnidae		jovem		1	P
Arachnida	Araneae	Ctenidae		jovem	1		
Arachnida	Araneae	Ochyroceratidae	*Ochyrocera*	sp.1	X	X	P/A
Arachnida	Araneae	Ochyroceratidae	*Speocera*	sp.1		X	E/P/A
Arachnida	Araneae	Pholcidae	*Mesabolivar*	sp.1		X	P
Arachnida	Araneae	Pholcidae	Ninetinae	sp.1		X	E/P
Arachnida	Araneae	Theridiidae		jovem	X	X	E
Arachnida	Araneae	Theridiosomatidae	*Plato*	sp.1	X	X	E/P
Arachnida	Opiliones	Escadabiidae		sp.2	X	X	E/A
Arachnida	Opiliones	Stygnidae	*Paraphareus*	sp.1	X	X	A
Arachnida	Pseudoscorpiones	Bochicidae		sp.1		X	A
Arachnida	Pseudoscorpiones	Chernetidae	*Spelaeochernes*	sp.1	X	X	E/P/A
Arachnida	Pseudoscorpiones	Chthoniidae		jovem		X	P
Chilopoda	Geophilomorpha	Ballophilidae	*Ityphilus*	sp.1		2	E/P
Chilopoda	Scolopendromorpha	Scolopocryptopidae	*Dinocryptops*	*miersii*	X		
Chilopoda	Scolopendromorpha	Scolopocryptopidae	*Tidops*	sp.1		1	E
Chilopoda	Scutigeromorpha	Pselliodidae		jovem		2	E/P
Diplopoda	Polydesmida	Chelodesmidae		jovem		3	A
Diplopoda	Polydesmida	Pyrgodesmidae		sp.1	X	X	A
Diplopoda	Spirostreptida	Pseudonannolenidae	*Pseudonannolene*	sp.1	X	9	A
Entognatha	Collembola	Cyphoderidae		sp.1		X	A
Entognatha	Collembola	Isotomidae		sp.1		X	A
Entognatha	Collembola	Sminthuroidea		sp.2	X	X	A
Entognatha	Diplura	Campodeidae		sp.1	X	X	E/P
Insecta	Blattodea	Blattellidae		jovem	X	4	P
Insecta	Coleoptera	Chrysomelidae		sp.4		X	P
Insecta	Coleoptera	Elateridae		jovem	X		
Insecta	Coleoptera	Tenebrionidae	Alleculinae	sp.	X		
Insecta	Diptera	Cecidomyiidae	Cecidomyiinae	sp.		X	E
Insecta	Diptera	Dolichopodidae		sp.		X	A
Insecta	Diptera	Drosophilidae	*Drosophila*	sp.	X		
Insecta	Diptera	Psychodidae	Phlebotominae	sp.		X	P
Insecta	Diptera	Psychodidae	*Pintomyia*	*gruta*	X		
Insecta	Diptera	Psychodidae	*Sciopemyia*	*sordellii*	X		
Insecta	Diptera	Sciaridae		sp.		X	A
Insecta	Heteroptera	Reduviidae	Reduviinae	sp.		4	P
Insecta	Heteroptera	Reduviidae	*Zelurus*	sp.	X		
Insecta	Homoptera	Cixiidae		jovem	X	X	E/P
Insecta	Hymenoptera	Formicidae	*Camponotus*	sp.1	X	X	P/A
Insecta	Hymenoptera	Formicidae	*Gnamptogenys*	sp.1		X	E
Insecta	Hymenoptera	Formicidae	*Hypoponera*	sp.1	X	X	A
Insecta	Hymenoptera	Formicidae	*Odontomachus*	*haematodus*	X		
Insecta	Hymenoptera	Formicidae	*Odontomachus*	sp.1		X	P
Insecta	Hymenoptera	Formicidae	*Odontomachus*	sp.2		X	P
Insecta	Hymenoptera	Formicidae	*Pachycondyla*	sp.1		X	A
Insecta	Hymenoptera	Formicidae	*Pachycondyla*	sp.2		X	E/P

Insecta	Hymenoptera	Formicidae	*Paratrechina*	sp.	X		
Insecta	Hymenoptera	Formicidae	*Strumigenys*	sp.	X		
Insecta	Hymenoptera	Ichneumonidae		sp.2		X	A
Insecta	Isoptera	Termitidae	*Nasutitermes*	sp.	X	X	E/P/A
Insecta	Lepidoptera	Noctuoidea		sp.	X		
Insecta	Lepidoptera	Tineoidea		sp.	X	X	
Insecta	Neuroptera	Myrmeleontidae		jovem		X	P
Insecta	Orthoptera	Phalangopsidae	*Phalangopsis*	sp.	X	1	A
Insecta	Psocoptera	Psocomorpha		jovem	X		
Insecta	Psocoptera	Psyllipsocidae		jovem		X	P
Insecta	Thysanura	Ateluridae	Atelurinae	sp.	X	X	
Gastropoda	Pulmonata	Subulinidae	*Allopeas*	sp.	X		
Gastropoda	Pulmonata	Subulinidae	*Lamellaxis*	sp.	X		
Gastropoda	Pulmonata	Systrophiidae	*Happia*	sp.		X	P
Malacostraca	Isopoda	Philosciidae		sp.1		X	A
Malacostraca	Isopoda	Scleropactidae	*Circoniscus*	*buckupi*	X	X	A
Mammalia	Chiroptera	Phyllostomidae	*Carollia*	*perspicillata*		1	P/A
Mammalia	Chiroptera	Phyllostomidae	*Micronycteris*	*microtis*		5	P/A
Mammalia	Chiroptera			sp.	20		P/A
Mammalia	Rodentia	Cricetidae	*Rhipidomys*	sp.		2	P/A
